# Small-Molecule GLP-1 Receptor Agonists: A Promising Pharmacological Approach

**DOI:** 10.3390/medicina61111902

**Published:** 2025-10-23

**Authors:** Oana Cristina Șeremet, Ciprian Pușcașu, Corina Andrei, Georgiana Nițulescu, Cristina Elena Zbârcea, Octavian Tudorel Olaru

**Affiliations:** 1 Department of Pharmacology and Clinical Pharmacy, Faculty of Pharmacy, Carol Davila University of Medicine and Pharmacy, 6 Traian Vuia Street, 020945 Bucharest, Romania; oana.seremet@umfcd.ro (O.C.Ș.); corina.andrei@umfcd.ro (C.A.); cristina.zbarcea@umfcd.ro (C.E.Z.); 2 Department of Pharmaceutical Technology and Biopharmacy, Faculty of Pharmacy, Carol Davila University of Medicine and Pharmacy, 6 Traian Vuia Street, 020945 Bucharest, Romania; 3 Department of Pharmaceutical Botany and Cell Biology, Faculty of Pharmacy, Carol Davila University of Medicine and Pharmacy, 6 Traian Vuia Street, 020945 Bucharest, Romania; octavian.olaru@umfcd.ro

**Keywords:** small-molecule GLP-1 receptor agonists, type 2 diabetes, obesity

## Abstract

Glucagon-like peptide-1 receptor (GLP-1R) agonists are injectable peptide-based therapies that have become a focal point in the medical community due to their significant therapeutic efficacy in type 2 diabetes and obesity treatment. Recent advancements in medicinal chemistry have enabled the development of small-molecule GLP-1R agonists, presenting advantages such as oral administration, improved patient adherence, and cost-effectiveness. These compounds demonstrate promising efficacy in enhancing insulin secretion and promoting weight loss, in a similar way to peptide agonists. This narrative review focuses on the pharmacodynamic profiles and the current progress in clinical and preclinical research on small-molecule GLP-1R agonists. As this class of agents continues to evolve, it represents a compelling therapeutic alternative with the potential to reshape the treatment for metabolic disorders.

## 1. Introduction

The incretin effect, first described in the 1960s, refers to the observation that oral glucose administration elicits a significantly greater insulin response than intravenous glucose, despite identical plasma glucose concentrations [1,2,3]. This phenomenon is largely mediated by two peptides: glucagon-like peptide-1 (GLP-1), secreted from enteroendocrine L-cells in the distal ileum and colon, and glucose-dependent insulinotropic polypeptide (GIP), secreted from K-cells in the proximal small intestine [4,5,6].

GLP-1 and GIP regulate meal-related glycemic fluctuations by augmenting insulin secretion in a glucose-dependent manner, thereby preventing postprandial hyperglycemia. Beyond its direct insulinotropic effect, GLP-1 exerts several additional actions: it suppresses glucagon release, delays gastric emptying, reduces appetite and food intake, and may promote β-cell survival and proliferation [7,8]. These effects make GLP-1 a central regulator of both short-term glycemic control and long-term energy balance. In individuals with type 2 diabetes mellitus (T2DM) [9], the incretin effect is markedly impaired, with GLP-1 activity being preserved but diminished, and GIP activity being significantly reduced [10]. This impairment has positioned incretin-based therapies as an attractive strategy for restoring physiological glucose regulation. In parallel, recent expert perspectives highlight that dietary interventions, including Mediterranean, low-carbohydrate, high-fiber, and plant-based diets, alongside complementary approaches such as probiotics and synbiotics, can enhance insulin sensitivity through mechanisms involving GLP-1 receptor signaling, insulin receptor activation, and peroxisome proliferator–activated receptors (PPARs). These insights reinforce the importance of integrating nutritional strategies with pharmacological approaches in the management of type 2 diabetes [11]. Pharmacological interventions include peptide-based GLP-1 receptor (GLP-1R) agonists, which mimic endogenous GLP-1, but resist enzymatic degradation, and dipeptidyl peptidase-4 (DPP-4) inhibitors, which prolong the half-life of endogenous incretins [7,12].

## 2. Peptidic GLP-1R Agonists

The approved GLP-1R agonists are peptide-based molecules that mimic the action of endogenous GLP-1, enhancing glucose-dependent insulin secretion, suppressing glucagon release, delaying gastric emptying, and thereby improving glycemic control in patients with type 2 diabetes. Several agents have been approved over the past decade, including exenatide, dulaglutide, and semaglutide, which differ in duration of action and administration schedules, but share a favorable efficacy and safety profile. Recently, tirzepatide received regulatory approval as the first dual agonist of the GLP-1 and GIP receptors. (Table 1). These drugs not only provide glucose-lowering effects but also contribute to weight reduction and cardiovascular protection, making them an attractive therapeutic option for patients with type 2 diabetes and associated comorbidities [13,14,15].

### 2.1. Efficacy in Diabetes and Obesity

The efficacy of GLP-1R agonists in T2DM management is well established. By enhancing insulin secretion in a glucose-dependent manner and reducing glucagon release, these agents effectively lower glycated hemoglobin (HbA1c) with a low risk of hypoglycemia. GLP-1R agonists significantly reduce HbA1c and fasting plasma glucose, with tirzepatide and semaglutide showing the greatest effects (HbA1c reduction of up to 2.1% and 2.37%, respectively) [16].

Clinical studies and meta-analyses show that approved GLP-1R agonists (liraglutide, semaglutide, tirzepatide) induce a significant reduction in body weight in adults with obesity or overweight, both with and without diabetes. The effects vary depending on the compound and dosage with liraglutide (3 mg/day, −5.8% at 26 weeks), semaglutide (2.4 mg/week, −13.9% body weight at 68 weeks), and tirzepatide (15 mg/week, −17.8% body weight at 72 weeks). Other molecules (exenatide, dulaglutide) show moderate effects, between −2.8 and −4.3 kg [17]. These effects are mediated through delayed gastric emptying and increased satiety [18].

### 2.2. Cardioprotective, Neuroprotective, and Other Benefits

Clinical studies and meta-analyses show that GLP-1R agonists reduce the risk of major adverse cardiovascular events (MACE) by 10–14%, including cardiovascular mortality, nonfatal myocardial infarction, stroke and all-cause mortality, in patients with T2DM. The effects are more pronounced in patients with cardiovascular risk factors [19,20,21]. Also, in patients without T2DM but with established cardiovascular disease, overweight or obese, the SELECT trial demonstrated that once-weekly subcutaneous semaglutide 2.4 mg significantly reduced the risk of cardiovascular death, nonfatal myocardial infarction, and nonfatal stroke compared with placebo during a mean follow-up of 39.8 months [22]. Recent meta-analyses also suggest that GLP-1R agonists reduce kidney disease progression in individuals with T2DM or overweight/obesity regardless of chronic kidney disease (CKD) status and are associated with a lower incidence of all-cause mortality and major adverse kidney events in patients with T2DM and established CKD [23,24].

Thanks to these beneficial effects, semaglutide was approved by the Food and Drug Administration (FDA) in January 2025 for reducing the risk of CKD progression, end-stage renal failure, and cardiovascular death in adults with type 2 diabetes and chronic kidney disease (CKD). This approval is based on results from the FLOW trial, which demonstrated that semaglutide significantly lowers the incidence of major renal and cardiovascular events, representing an important advancement in the management of patients with diabetes and concomitant renal impairment [25].

GLP-1R agonists have demonstrated neuroprotective effects across a range of neurodegenerative disorders, with preclinical studies demonstrating efficacy in animal models of Alzheimer’s, Parkinson’s disease, stroke, and ischemia, likely mediated through a reduction in chronic inflammation, normalization of insulin and growth factor signaling, enhanced energy utilization, and mitochondrial protection. Also, early results from clinical trials in patients with Alzheimer’s or Parkinson’s disease indicate clear neuroprotective benefits [26,27].

In August 2025, the FDA approved subcutaneous semaglutide for the treatment of MASH (Metabolic Dysfunction-Associated Steatohepatitis), an advanced form of steatohepatitis associated with metabolic dysfunction, in adults with moderate to advanced liver fibrosis but without cirrhosis. The approval was based on interim results from the ESSENCE clinical trial, which demonstrated that 63% of participants treated with semaglutide 2.4 mg weekly experienced resolution of MASH without progression of liver fibrosis, compared to 34% in the placebo group, and 37% of treated participants showed improvement in liver fibrosis without worsening of MASH, versus 22% in the placebo group.

### 2.3. Limitations of GLP-1R Agonist

Despite their benefits, GLP-1R agonists have notable limitations. High cost is the most significant barrier, with modeling studies showing that prices must decrease by at least 70% for them to be considered cost-effective as a first-line therapy. These agents must be stored between 2–8 °C and must never be frozen, since freezing can cause protein denaturation and loss of efficacy. The need for strict cold chain maintenance further increases the cost of GLP-1R agonists, as specialized storage, transport, and monitoring requirements add to the overall economic burden of these therapies.

Most agents are injectable, which may affect patient acceptance due to injection discomfort and the need for pen devices [28]. Oral semaglutide provides a noninvasive alternative; however, it is currently approved only for T2DM and lacks regulatory authorization for weight management, cardiovascular, renal, or hepatic indications.

The most common adverse reactions (AEs) to GLP-1R agonists are gastrointestinal (GI) events. Nausea, vomiting, diarrhea, and constipation are the most frequently observed, occurring in 20–50% of patients, especially during treatment initiation or dose escalation [29]. All GLP-1R agonists may increase the risk of acute pancreatitis, with stronger signals reported for liraglutide and exenatide. Patients at risk of pancreatitis should avoid this class of drugs [30]. Reports of medullary and papillary thyroid carcinoma have been observed with GLP-1R agonists, though a definitive causal relationship in humans remains unconfirmed, making regular monitoring advisable [31].

## 3. The GLP-1 Receptor: Structure, Signaling, and Implications for Non-Peptidic Agonists

GLP-1R is widely expressed, with particularly high levels in the pancreatic islets, where it is predominantly localized to β-cells and mediates insulinotropic actions. Beyond the pancreas, GLP-1R is present in multiple tissues including the GI tract, heart, kidney, lung, and central nervous system, reflecting its diverse physiological roles. In the brain, GLP-1R expression is enriched in the hypothalamus and brainstem, regions involved in appetite regulation and autonomic control. Within β-cells, the receptor is primarily localized to the plasma membrane, though evidence also supports receptor trafficking to endosomal compartments, which may contribute to sustained intracellular signaling. This broad distribution underscores the pleiotropic effects of GLP-1 and provides the anatomical basis for the therapeutic benefits of GLP-1R agonists in metabolic and cardiovascular disease [32,33].

Upon ligand binding, GLP-1R primarily couples to Gαs, leading to cAMP accumulation and activation of downstream effectors such as protein kinase A (PKA) and the exchange protein directly activated by cAMP 2A (EPAC2A), which are central to its insulinotropic actions in pancreatic β-cells. In addition to this canonical pathway, GLP-1R can engage alternative G proteins, including Gαi/o and Gαq, thereby recruiting signaling cascades involving extracellular signal-regulated kinases 1 and 2 (ERK1/2) and protein kinase B (PKB/Akt) [34,35].

The interaction between GLP-1R and the G protein also trigger G protein-coupled receptor kinases (GRKs), which detect the active conformation of GLP-1R and phosphorylate it, thereby enabling the recruitment of β-arrestin 1 and -2. By binding to activated receptors, β-arrestins terminate G protein coupling and regulate receptor internalization and trafficking. Moreover, receptor–β-arrestin complexes function as independent signaling hubs, regulating multiple intracellular pathways [36,37].

GLP-1R is a member of the class B family of G protein-coupled receptors (GPCRs). GPCRs are generally grouped into several classes, such as class A (rhodopsin-like), class B (secretin-like), and class C (metabotropic glutamate/pheromone receptors). Class B receptors are characterized by the presence of a large extracellular N-terminal domain (ECD) essential for binding peptide ligands such as GLP-1 [38].

Structurally, GLP-1R combines this conserved ECD with a seven-helix transmembrane (TM) domain, organized into seven α-helices joined by three extracellular and three intracellular loops. Within the ECD, several conserved amino acids—Asp67, Trp72, Pro86, Gly108, and Trp110—help stabilize its structure [39].

Endogenously, GLP-1 is secreted in two closely related forms: GLP-1(7–36) amide, which terminates with an amidated Arg36, and GLP-1(7–37), the glycine-extended or “acid” form. Both isoforms interact with GLP-1R [40,41]. In the activation process, the C-terminal region of GLP-1 engages the receptor ECD, after which the N-terminal segment inserts into the TM domain. These interactions allow the N-terminus of GLP-1 to access a deep pocket within the TMs bundle, thereby inducing conformational rearrangements of the helical bundle. A key feature of this activation is the outward displacement of TM6, which opens the intracellular cavity and enables coupling of the receptor to the G protein [42,43]. The His7–Ala8–Glu9–Gly10 motif of N-terminus of GLP-1 constitutes a critical determinant for receptor binding and activation. Substitution studies demonstrated that position 7 requires a small aromatic side chain, whereas position 8 only accommodates small residues, reflecting steric and conformational constraints of the binding pocket [44].

Therapeutic peptide analogues such as semaglutide exploit this requirement by introducing 2-aminoisobutyric acid (Aib) at position 8, which both preserves receptor compatibility and confers resistance to dipeptidyl peptidase-4 (DPP-4) cleavage [45]. At position 9, negatively charged or size-compatible residues are tolerated, while positively charged substitutions markedly reduce affinity. Gly10 is indispensable, with replacement consistently impairing receptor interaction and potency [44]. The N-terminal region is therefore essential for receptor activation, and removal of His7–Ala8 by DPP-4—producing GLP-1(9–36) amide or GLP-1(9–37)—abolishes the peptide’s ability to activate GLP-1R [46].

Designing small non-peptidic ligands that mimic large, flexible peptide agonists is inherently difficult. Unlike peptides, which can present multiple hydrogen-bond donors, acceptors, and amphipathic motifs across a broad surface, small molecules are limited in both surface area and chemical diversity [47]. Boc5 was the first non-peptidic agonist of GLP-1R, demonstrating that small molecules can mimic incretin activity. Pharmacologically, it induces cAMP accumulation to levels comparable to native GLP-1 and stimulates insulin secretion both in vitro and in vivo models [48].

The non-peptidic agonists interact with distinct domains of the GLP-1R. The majority are orthosteric ligands interact with the binding pocket, directly mimicking the activity of endogenous GLP-1. In contrast, allosteric ligands function as positive allosteric modulators (PAMs), occupying alternative binding sites to potentiate receptor responsiveness to the native hormone [37]. Some of the orthosteric ligands, like danuglipron, orforglipron, act as biased agonist of the GLP-1R that selectively activates specific signaling pathways of the receptor, such as the G protein pathway, responsible for the therapeutic effect, over others, like β-arrestin recruitment, to provide therapeutic benefits with fewer side effects [49].

The aim of this narrative review is to critically evaluate the current state of research and development on non-peptidic GLP-1R agonists, highlighting their pharmacological properties and therapeutic potential. The review seeks to summarize mechanistic insights into GLP-1R activation and assess preclinical and clinical evidence regarding efficacy, safety, and pharmacokinetic profiles of emerging non-peptidic GLP-1R agonists.

## 4. Materials and Methods

A comprehensive literature search was conducted to identify relevant publications on small-molecule GLP-1R agonists. The search was performed in PubMed using the keywords “small molecule GLP-1 receptor agonists” and “non-peptidic GLP-1 receptor agonists”. The specific names of non-peptidic GLP-1R agonist molecules were also used as keywords to broaden the search strategy. To capture ongoing or recently completed clinical trials, the same search terms were applied in the ClinicalTrials.gov database.

In addition to peer-reviewed publications and registered trials, we also included official press releases and communications from pharmaceutical manufacturers, which provided complementary information regarding the clinical development, efficacy outcomes, and safety profiles of emerging compounds.

The search covered publications and records available up to August 2025.

## 5. Small-Molecule Agonists of GLP-1R

The pursuit of orally bioavailable GLP-1R agonists represents a significant advancement in T2DM and obesity pharmacotherapy, aiming to replace the inconvenience of injectable incretin therapies. Over the past decade, multiple non-peptidic small molecules have been developed, targeting the GLP-1R pathway with varying degrees of potency, signaling bias, and clinical outcomes.

This section offers a comprehensive review of key non-peptidic GLP-1R agonists, ranging from discontinued compounds to ongoing clinical candidates, highlighting their pharmacodynamics, pharmacokinetics, therapeutic efficacy, safety, and developmental status.

A summary of the key characteristics, preclinical findings, and clinical outcomes of small-molecule GLP-1R agonists is presented in Table 2, while detailed descriptions of each compound are provided in the subsequent sections.

### 5.1. Boc5

Boc5 was the first non-peptidic GLP-1R agonist, identified via high-throughput CRE (cAMP response element)-luciferase screening targeting rat GLP-1R. Despite violating all Lipinski and Veber rules for oral drug-likeness, it remains a foundational molecule in non-peptidic GLP-1R agonist development [50,51]. Pharmacokinetic studies reveal that Boc5 has poor oral bioavailability, requiring high doses to achieve therapeutic plasma levels [49,51].

In preclinical studies, plasma concentrations were virtually undetectable after oral administration at 20 mg/kg in rats and 250 mg/kg in mice. In contrast, both intraperitoneal (ip) and intravenous (IV) dosing produced measurable plasma levels. These routes also prolonged t½, reaching 35.4 h in rats and 12.1 h in mice after ip dosing, and 41.7 h and 8.71 h, respectively, after IV dosing. Boc5 showed moderate plasma protein binding (~36.2%) and high metabolic stability in liver and intestinal microsomes. Neither CYP (cytochrome P450) nor UGT (uridine 5′-diphospho-glucuronosyltransferase) enzymes were involved in its metabolism. Instead, Boc5 underwent ester hydrolysis primarily mediated by human serum albumin. This mechanism resulted in species-dependent plasma t½ values: 23.5 h in humans, 31.8 h in rats, and 83.1 h in pigs [51,52,53].

Pharmacodynamically, Boc5 is a full agonist in CRE-luciferase assays but only a partial agonist in cAMP (cyclic adenosine monophosphate) accumulation (~30% maximal effect) [50,54]. It shows weak activity in ERK (extracellular signal-regulated kinases) 1/2 phosphorylation (19%) and Ca^2+^ mobilization (22%), and does not recruit β-arrestin 1 or 2. This profile suggests biased signaling with reduced receptor desensitization [50,54].

In diabetic rodent models, Boc5 demonstrated marked antidiabetic efficacy. Chronic administration (oral or IP) reduced blood glucose, HbA1c (glycated hemoglobin), and body weight [50,52,55]. Acute administration suppressed food intake in a dose-dependent manner, an effect reversed by exendin (9–39), confirming GLP-1R specificity [48].

Additional studies showed that Boc5 enhanced glucose-dependent insulin secretion, improved insulin sensitivity, reduced fat mass, delayed gastric emptying, and promoted satiety [52]. In diet-induced obese mice, it reduced body weight and food intake, leading to decreased fat mass, adipocyte hypertrophy, and lipid accumulation. These metabolic improvements were accompanied by enhanced insulin sensitivity, normalization of β-cell mass, and increased glucose uptake and lipolysis in isolated adipocytes. Additionally, Boc5 improved lipid profiles and restored leptin/adiponectin balance [55]. In vitro, Boc5 protected β-cell lines under oxidative, cytokine, or lipotoxic stress but did not induce β-cell proliferation under basal conditions [56].

Boc5 did not progress to clinical trials, most likely because of its low oral bioavailability, less favorable pharmacokinetics, and difficulties in achieving consistent systemic exposure, which together limited its therapeutic potential despite encouraging preclinical results.

### 5.2. TT-OAD2

TT-OAD2 is a partial, allosteric GLP-1R agonist with biased signaling. In HEK293 (human embryonic kidney 293) cells, TT-OAD2 showed limited orthosteric probe displacement, activated the cAMP/PKA protein kinase A (PKA) pathway selectively, and showed minimal Ca^2+^ and ERK1/2 activation at high doses. It did not recruit β-arrestin 1, confirming its signaling bias [57]. In GLP-1R humanized mice, TT-OAD2 exhibited insulinotropic effects [57,58] TT-OAD2 was not advanced to clinical studies.

### 5.3. TTP-273

TTP273 is an analogue from the same class as TT-OAD2, developed by vTv Therapeutics for the management of T2DM [59].

In in vitro experiments, TTP-273 demonstrated strong potency and high selectivity as a GLP-1R agonist, eliciting a concentration-dependent increase in receptor activation for both human and murine GLP-1R. Measurements of cAMP accumulation revealed significant activity exclusively at GLP-1R, with no relevant activation of closely related receptors, highlighting the compound’s receptor specificity [59,60].

Preclinical studies show a dose-dependent reduction in plasma glucose during oral glucose tolerance tests (GTT) in mice. In ob/ob (leptin-deficient) mice, 14 days of treatment improved glycemic control, reduced food intake, and slowed body weight gain. Notably, appetite suppression occurred only after oral dosing, suggesting involvement of hepatoportal GLP-1R activation and vagus nerve–mediated neuroendocrine pathways. Immunohistochemistry revealed elevated c-fos expression in select brain regions, despite only trace brain levels of the compound, indicating that central effects are likely indirect rather than due to CNS penetration [59,60].

From a pharmacokinetic standpoint, TTP-273 demonstrates a rapid absorption profile, achieving peak plasma concentrations within approximately 2h, and is characterized by a mean terminal elimination t½ of roughly 6 h [61].

A Phase IIa, 12-week trial (NCT02653599) showed that TTP-273 at 150 mg once daily reduced HbA1c by 0.86% versus a placebo, while the same dose twice daily achieved a 0.71% reduction; the placebo group recorded a 0.15% increase. Modest weight loss was observed (0.9 kg once daily; 0.6 kg twice daily), without a clear dose–response effect. Safety analysis indicated lower nausea rates in both active arms compared with placebo and no vomiting, suggesting a favorable GI tolerability profile [62,63,64].

In the post hoc analysis, systolic blood pressure decreased on average by 17 mmHg in the once-daily group and by 12 mmHg in the twice-daily group, compared with a 6 mmHg reduction in the placebo group. No significant variations were observed in diastolic blood pressure or heart rate [65].

### 5.4. Danuglipron

Danuglipron (PF-06882961) is a potent, orally active small molecule targeting GLP-1R, with an effective half-maximal concentration (EC_50_) of 13 nM for cAMP accumulation. It acts as a full agonist for cAMP signaling but only partially activates Ca^2+^ mobilization, ERK1/2 phosphorylation, and β-arrestin pathways [66,67].

Preclinical models showed superior glucose-lowering and anorexigenic effects compared to exendin-4 or liraglutide [67]. In a GLP-1R humanized knock-in mouse model, subcutaneous administration (30 mg/kg) improved glucose tolerance, increased plasma insulin levels, and reduced food intake at multiple timepoints post-dose [66]. Pharmacokinetic studies showed moderate to high plasma clearance after IV dosing in rats (57.3 mL/min/kg) and cynomolgus monkeys (13.8 mL/min/kg), with short t½ values (1.1 and 1.9 h, respectively). The oral bioavailability of the Tris salt form, formulated with tris(hydroxymethyl)aminomethane to improve solubility and stability, was low to moderate but dose-dependent, and adequate for in vivo testing when combined with methylcellulose and Tween 80. Subcutaneous dosing (2.9 mg/kg for two days) also significantly reduced food intake in monkeys [67].

In healthy volunteers, a first-in-human, placebo-controlled, dose-escalation Phase I study (NCT03309241) demonstrated that oral doses from 3 to 300 mg were well tolerated. Plasma exposure increased dose-proportionally (t½: 4.3–6.1 h; Tmax (time to maximum concentration): 2–6 h), and food intake had minimal impact on pharmacokinetics. The most frequent adverse events (AEs) were mild nausea, vomiting, and appetite loss, with no hypoglycemia or serious AEs reported [67].

Another Phase I open-label study (NCT04616027) assessed pharmacokinetics and safety in participants with varying degrees of renal impairment. Danuglipron exposure and clearance were unaffected by renal function, and <1% of the dose was excreted unchanged. No clinically meaningful safety concerns were identified, suggesting no need for dose adjustment in T2DM patients with renal impairment [68].

In Japanese patients with T2DM (NCT04552470), danuglipron (40–120 mg twice daily for 8 weeks) produced dose-proportional increases in exposure and statistically significant reductions in HbA1c (up to −1.41%), fasting plasma glucose (−40.9 mg/dL), mean daily glucose (−67.9 mg/dL), and body weight (up to −1.87 kg). GI-related AEs were frequent, but mostly mild [69].

In a 28-day phase Ib trial (NCT03538743), danuglipron produced dose-dependent reductions in HbA1c (>0.8% at multiple doses), fasting plasma glucose (up to −76.9 mg/dL), and body weight (−4.4 kg at 70 mg twice daily and −7.2 kg at 120 mg twice daily). The incidence of GI AEs increased with dose, peaking at 100% for 120 mg twice daily. Dose titration improved tolerability, and only one case of mild, non-fasting hypoglycemia was reported [66].

A Phase IIb trial (NCT03985293) involving 411 adults with T2DM treated with danuglipron (2.5–120 mg twice daily for 16 weeks) showed significant, dose-dependent reductions in HbA1c (up to −1.16%), fasting glucose (−33.24 mg/dL), and body weight (up to −4.17 kg). Nausea, vomiting, and diarrhea were the most common AEs [70].

Another Phase IIa study (NCT04617275) evaluated dose-escalation schemes up to 200 mg twice daily. In T2DM patients, danuglipron reduced HbA1c (−1.04% to −1.57%), fasting glucose (−23.3 to −53.9 mg/dL), and body weight (−1.93 to −5.38 kg). GI AEs were dose-related, with discontinuation rates up to 72.7% [71].

In a 26/32-week Phase IIb study (NCT04707313) in obese individuals without T2DM, danuglipron (40–200 mg twice daily) resulted in placebo-adjusted weight reductions ranging from −5.0% to −12.9%. Discontinuation occurred in 38% of participants, mainly due to GI AEs, especially at higher doses [72].

Beyond its antidiabetic properties, danuglipron also exhibited cardioprotective effects in a murine model of pressure overload-induced cardiac hypertrophy and fibrosis. Oral administration (1 mg/kg/day for 8 weeks) attenuated pathological cardiac remodeling via AMPK (AMP-activated protein kinase) phosphorylation and HSP70 (heat shock protein 70) upregulation. These effects were abolished in AMPKα2-deficient mice [73].

Despite its promising glycemic, metabolic, and cardioprotective effects, Pfizer formally discontinued the clinical development of danuglipron in April 2025. The decision followed observations of asymptomatic liver enzyme elevations during a Phase II dose-optimization trial. Although the abnormalities resolved upon treatment cessation, Pfizer halted both twice-daily and once-daily formulations due to safety concerns and internal portfolio reprioritization [74].

### 5.5. Orforglipron

Orforglipron (LY3502970 or OWL833) is a small-molecule compound that acts as a partial agonist of GLP-1R [58].

In vitro experiments using HEK293 cells engineered to express human GLP-1R demonstrated that orforglipron strongly enhanced cyclic adenosine monophosphate (cAMP) generation via Gs protein-mediated pathways, without inducing β-arrestin recruitment. This biased agonism profile is linked to sustained receptor responsiveness and may attenuate certain (AEs) typically associated with full GLP-1R agonists [75,76].

In in vivo studies, oral administration in transgenic mice expressing human GLP-1Rs significantly improved glucose tolerance. Pharmacokinetic/pharmacodynamic analyses indicated that low receptor occupancy was sufficient to achieve maximal glucose-lowering activity [76]. In a human GLP-1R–expressing murine model, oral dosing (0.1–10 mg/kg) in overnight-fasted mice produced marked glycemic reductions 5 h after treatment during IP GTT. Peak pharmacodynamic effects occurred at relatively low plasma concentrations: 24 ± 8 nmol/L (0.1 mg/kg), 205 ± 18 nmol/L (1 mg/kg), and 1257 ± 387 nmol/L (10 mg/kg). Comparative testing with exenatide showed similar glucose-lowering efficacy, while no activity was detected in Glp1R-deficient controls [75].

In cynomolgus monkeys, oral OWL833 reduced food intake by ~25% and ~45% in two separate regimens, achieving anorexic effects comparable to subcutaneous exenatide, thus supporting translational relevance across species [77]. In S33W GLP-1R mutant rats, which display heightened sensitivity to non-peptidic agonists, chronic treatment with orforglipron induced weight loss comparable to that achieved with semaglutide, with sustained efficacy even after crossover from the injectable peptide to oral orforglipron [76].

In early clinical studies, orforglipron displayed pharmacokinetics compatible with once-daily oral dosing [78,79]. In healthy participants (Phase Ia), the terminal elimination t_1_/_2_ was ~25–35 h after single doses (0.3–6 mg) and extended to ~48–68 h after 28 days of repeated daily dosing (2–24 mg) [78]. In individuals with T2DM (Phase Ib), t_1_/_2_ ranged from ~29 to 49 h over 12 weeks of treatment with final doses between 9 and 45 mg [79]. Systemic exposure increased in an approximately dose-proportional manner, with median Tmax between 4–12 h in healthy volunteers and 4–8 h in patients with T2DM [78,79].

Another Phase I trial in healthy adults evaluated the effect of food on orforglipron pharmacokinetics, showing 17–24% lower AUC and Cmax in the fed versus fasted state, with no impact on t_1_/_2_ or Tmax [80].

Furthermore, several Phase I trials of orforglipron (NCT06440980, NCT06370728, NCT06704763, NCT06186622)—including bioequivalence studies in overweight and obese participants and multiple drug–drug interaction investigations—have been completed; however, no results have yet been publicly disclosed [81,82,83,84].

From a pharmacodynamic perspective, orforglipron produced rapid and sustained improvements in glycemic control and body weight. In healthy participants, 2 h OGTT glucose decreased significantly on Day 1 at all doses (0.3–6 mg; −27.0 to −79.3 mg/dL). Higher-dose groups maintained reductions through Day 28, although the response was not strictly linear. These effects correlated with delayed gastric emptying and progressive, dose-dependent weight loss, reaching −4.8 to −5.4 kg at doses ≥6 mg versus −2.4 kg with placebo [78]. In T2DM, 12 weeks of treatment (9–45 mg) reduced HbA_1_c compared with placebo by −1.02% to −1.38%, lowered fasting plasma glucose by up to −3.0 mmol/L, and achieved body-weight loss of approximately −4 to −6 kg in most dose groups (except 21 mg) [79].

These early Phase I findings were subsequently corroborated and extended in longer-term Phase II trials, which demonstrated significant and sustained improvements in glycemic control and body weight [85,86]. A 26-week multicenter trial in adults with T2DM, managed by lifestyle measures with or without metformin, showed that daily orforglipron ≥12 mg outperformed both placebo and dulaglutide. HbA_1_c decreased by 2.10% with orforglipron, compared with 0.43% with placebo and 1.10% with dulaglutide. Body weight reductions were −10.1 kg, −2.2 kg, and −3.9 kg, respectively, over the 26-week period [85]. In a separate 36-week study enrolling individuals with obesity or overweight and at least one weight-related comorbidity, but without T2DM, orforglipron treatment resulted in dose-dependent reductions in body weight. At week 26, weight loss ranged from 8.6% to 12.6%, increasing to 9.4% to 14.7% by week 36, compared with reductions of 2.0% and 2.3% in the placebo group, respectively. By the end of the study, 46% to 75% of participants receiving orforglipron achieved a weight reduction of at least 10%, compared with 9% in the placebo group, accompanied by improvements in cardiometabolic risk markers [86].

Following the positive outcomes in Phase II, orforglipron progressed to Phase III trials to confirm its efficacy, safety, and long-term benefits in broader patient populations. The ACHIEVE-1 phase III trial demonstrated that 40 weeks of once-daily orforglipron (3–36 mg) therapy in adults with early T2DM led to significant reductions in HbA1c (−0.83 to −1.07 percentage points vs. placebo). Treatment also induced dose-dependent weight loss (−4.5% to −7.6% vs. −1.7% with placebo) [87]. The Phase III ATTAIN-1 trial evaluated orforglipron in adults with obesity over a 72-week period. Treatment with the highest tested dose (36 mg) resulted in a mean weight loss of 12.4% compared with 0.9% in the placebo group. A reduction in body weight of at least 10% was achieved by 59.6% of participants, while 39.6% achieved a loss of 15% or more [88].

Currently, active phase III trials are evaluating orforglipron in obesity and type 2 diabetes associated with comorbid conditions such as hypertension and obstructive sleep apnea [89,90,91,92].

Across phase I–III trials, the compound showed a favorable safety profile. Gastrointestinal events (nausea, vomiting, constipation, decreased appetite) were the most common, typically mild to moderate and occurring mainly during initiation or dose escalation. Hypoglycemia was rare, and no severe episodes were reported, with overall good tolerability throughout clinical development [78,79,85,86,87,88].

### 5.6. HRS-7535

HRS-7535 is a novel oral GLP-1R agonist evaluated in multiple clinical trials for T2DM and obesity. To date, no information has been disclosed regarding its receptor-binding characteristics or preclinical evaluation.

In a Phase I trial (NCT05347758) in healthy participants, the safety, tolerability, pharmacokinetics, and pharmacodynamics of HRS-7535 were assessed across SAD, food effect, and multiple-ascending dose (MAD) cohorts. The most common AEs were nausea and vomiting. In the MAD part, daily titration up to 120 mg for 28 days led to a mean body weight reduction of 4.38 kg versus 0.8 kg with placebo. The pharmacokinetic profile was dose-proportional, with a Tmax of 5.98–10.98 h and t½ of 6.48–8.42 h [93].

In a 16-week Phase II trial (NCT05759897), once-daily HRS-7535 (15–90 mg) significantly reduced HbA_1_c (−0.94% to −1.57%) and improved fasting/postprandial glucose, weight, and waist circumference in patients with T2DM on metformin. The treatment was generally well tolerated, with mostly mild to moderate GI AEs, low rates of hypoglycemia (5.8%, none severe), and modest increases in pulse rate and pancreatic enzymes [94].

In a 36-week Phase II trial (NCT06250946) involving adults with obesity but without T2DM, once-daily HRS-7535 (30–180 mg) led to significant weight loss. At week 26, participants receiving 180 mg lost 6.87% more weight than placebo, increasing to 9.5% by week 36. Moreover, 35.4% of them achieved ≥10% weight loss versus 6.5% in the placebo group. AEs, mainly GI (nausea, diarrhea, vomiting), were common during dose titration but generally mild. Liver enzymes remained stable, and discontinuation due to side effects was low (2.1%) [95].

### 5.7. ECC5004

ECC5004 (AZD5004) is a non-peptidic, orally bioavailable GLP-1R agonist that was originally developed by Eccogene (Shanghai, China) and subsequently licensed to AstraZeneca (Cambridge, UK) for continued clinical development [96].

In cell-based assays, ECC5004 exhibited high binding affinity for the human GLP-1R (IC_50_ (inhibitory concentration 50%) = 2.4 nM) and acted as a full agonist for cAMP accumulation in HEK293 cells (EC_50_ = 2.1 nM). In contrast, it functioned as a partial agonist in CHO (Chinese hamster ovary)-K1 cells (Emax (maximum effect) = 30%; EC_50_ = 45 nM), with no detectable recruitment of β-arrestin-2 or receptor internalization, indicating a G-protein biased signaling profile. Additionally, in human pancreatic β-cell line, ECC5004 significantly potentiated glucose-stimulated insulin secretion with an EC_50_ of 5.9 nM [96].

In non-human primates, IV ECC5004 (0.5 mg/kg) showed a t½ of 1.72 h and dose-dependent effects on insulin secretion and glucose clearance (EC_50_ = 0.022 nM). A 9-month toxicology study indicated reduced body weight gain but no major safety concerns, establishing a non-observed AE level of 50 mg/kg/day [96].

In a first-in-human Phase I study (NCT05654831), single ascending doses (SAD, 1–300 mg) of ECC5004 were administered to healthy volunteers. The compound was generally well tolerated, with most AEs limited to mild GI symptoms (nausea and vomiting). Dose-proportional PK was observed at ≥25 mg, and the 300 mg dose reduced glucose AUC_0–2_h by up to 38%. In the multiple ascending dose (MAD) part, patients with T2DM received ECC5004 (5–50 mg) for 28 days. Treatment was well tolerated overall, with no serious AEs. Two participants discontinued due to transient QTc prolongation and elevated liver enzymes, both of which resolved. At the 50 mg dose, fasting plasma glucose decreased by 76.6 mg/dL and glucose AUC_0–4_h by 590.2 h·mg/dL. ECC5004 also induced a 5.76% body weight reduction versus 3.52% with placebo [96].

ECC5004 is currently being investigated as part of AstraZeneca’s metabolic pipeline. As announced by the company in October 2024, a global Phase II clinical trials are underway to evaluate its efficacy, safety, and dose–response profile in patients with obesity and T2DM [97,98,99].

### 5.8. CT-996

CT-996 is an orthosteric agonist of GLP-1R, designed with a signaling bias, and it predominantly stimulates cAMP production while eliciting minimal β-arrestin engagement [100]. In GLP-1R–expressing cell-based functional assays, CT-996 exhibited high potency in cAMP production, with an EC_50_ of 0.049 nM, closely approaching that of native GLP-1 (0.009 nM). In contrast, CT-996 demonstrated markedly reduced β-arrestin 2 recruitment (EC_50_ = 384 nM vs. 16 nM for GLP-1) and substantially weaker receptor internalization (EC_50_ = 106 nM vs. 0.55 nM for GLP-1). These findings indicate that CT-996 is a highly potent, cAMP-biased GLP-1R agonist that preferentially engages G protein-mediated signaling over β-arrestin–dependent pathways [101,102].

In vivo studies confirm the translational potential of CT-996, showing rapid and sustained improvements in glycemic control, insulin secretion, and body weight regulation in both transgenic mice and obese cynomolgus monkeys [103].

Phase I clinical data indicate that CT-996 is well tolerated in adults, with overweight and obese participants, with or without T2DM, showing mainly mild-to-moderate GI AEs. Pharmacokinetic evaluation showed that food intake, whether fasting or following a high-fat meal, had minimal impact on systemic exposure, supporting flexible once-daily dosing. CT-996 displayed a Tmax of approximately 8–9.6 h and an elimination t½ of 17.1–21.8 h. Participants underwent a structured dose-escalation regimen comprising 10 mg for 3 days, 30 mg for 4 days, 50 mg for 7 days, 80 mg for 7 days, and 120 mg for 7 days. At Day 29, this titration protocol resulted in a significant reduction in mean body weight compared with placebo (−7.3% vs. −1.2%) [102,104,105].

Based on these findings, CT-996 is advancing into Phase II clinical trials, planned to commence in 2025, to further evaluate its efficacy in glycemic control, weight reduction, and long-term weight maintenance.

### 5.9. Aleniglipron

Aleniglipron (GSBR-1290) is a potent agonist of the GLP-1R Gαs–cAMP signaling pathway without detectable β-arrestin recruitment, indicating that it functions as a fully biased agonist [106].

Its effect on insulin secretion was assessed in a functional human pancreatic beta cell line, where aleniglipron induced insulin release in a dose-dependent manner. The in vivo efficacy of aleniglipron was further evaluated in nonhuman primates. In an acute IV-GTT, a single dose of aleniglipron markedly stimulated insulin secretion and enhanced glucose clearance. In a repeated dosing study, oral daily administration for seven days produced a robust increase in insulin secretion and glucose clearance during an IV-GTT test, along with a dose-dependent reduction in food intake and body weight [106].

The safety and tolerability of an MAD (5–90 mg) of aleniglipron, along with its effects on body weight, were evaluated in a Phase Ib study in healthy overweight volunteers over a 4-week period. In Phase IIa, aleniglipron was further assessed for safety, tolerability, HbA1c, glucose levels, and body weight in patients with T2DM, as well as in healthy overweight volunteers.

Aleniglipron was generally well tolerated across studies, with most AEs being mild to moderate GI events, consistent with the GLP-1RA class, and no serious drug-related adverse events. In a Phase Ib study in healthy overweight volunteers, body weight decreased significantly over 4 weeks (placebo-adjusted maximum −4.9%, *p* = 0.013). In Phase IIa, treatment reduced HbA1c (45 mg: −1.01%, *p* = 0.008; 90 mg: −1.02%, *p* = 0.001), body weight (45 mg: −3.51%, *p* = 0.0019; 90 mg: −3.26%, *p* = 0.0013), and plasma glucose (45 mg: −2.70 mmol/L, *p* = 0.01; 90 mg: −2.50 mmol/L, *p* = 0.0008) at Day 84 in patients with T2DM. In participants with obesity, placebo-adjusted body weight was significantly reduced by Day 56 (120 mg: −4.74%, *p* < 0.0001) [107].

Currently, a Phase IIb dose-range finding study (ACCESS, NCT06693843) is evaluating the efficacy and safety of multiple doses of aleniglipron (GSBR-1290) in participants with obesity or overweight who have at least one weight-related comorbidity [108].

### 5.10. ID110521156

ID110521156 is a small-molecule GLP-1R agonist designed for oral bioavailability. It demonstrated direct binding to the GLP-1R in a surface plasmon resonance assay and induced a dose-dependent increase in intracellular cAMP levels in CHO cells expressing the human GLP-1R, with maximal efficacy similar to that of GLP-1 (7–36) amide. While partial agonism was observed in the recruitment of β-arrestin, no effects were detected on intracellular Ca^2+^ levels, mitogen-activated protein kinase phosphorylation, or GLP-1R internalization.

In vivo, ID110521156 lowered plasma glucose, enhanced insulin secretion, and reduced food intake and body weight in diabetic monkeys. In cynomolgus monkeys, it showed a t½ of 3.7–4.9 h and oral bioavailability of 18–32%. A 28-day toxicity study confirmed a 100-fold safety margin, with no cardiovascular, respiratory, neurobehavioral, or genotoxic effects [109].

Following the favorable nonclinical results, a first-in-human study was initiated in the Republic of Korea (NCT06063291). This randomized, double-blind, placebo-controlled SAD study enrolled 24 healthy subjects to assess the safety, tolerability, and pharmacokinetics of ID110521156. A separate food effect study used a crossover design to evaluate the impact of food on drug absorption. Fourteen subjects reported 32 treatment-emergent AEs, mostly mild to moderate GI events such as nausea and vomiting. No serious AEs occurred. Food had minimal impact on systemic exposure, although Cmax was reduced by 36% after a high-fat meal [110].

To further investigate the compound’s pharmacological profile, a Phase I MAD study (NCT06635226) is currently underway in healthy adults in the Republic of Korea. This randomized, double-blind, placebo-controlled trial aims to evaluate the safety, tolerability, pharmacokinetics, and pharmacodynamics of ID110521156 following repeated oral administration [111].

### 5.11. XW-014

XW-014 is an oral small-molecule GLP-1R agonist developed by Hangzhou Sciwind Biosciences (Hangzhou, China) that was demonstrated to be a potent antagonist of cAMP pathway activation in HEK293 cells expressing GLP-1R from both humans and cynomolgus monkeys [13].

In mice engineered to express human GLP-1R, oral administration of XW-014 improved blood glucose AUC, after IV-GTT administration in a dose-dependent fashion. Comparable benefits were observed in cynomolgus monkeys after oral treatment. In addition, in diet-induced obese mice, a 15-day dosing regimen reduced blood glucose, food intake, and body weight [13].

It is currently being evaluated in a Phase I clinical trial (NCT05579314), which is a randomized, double-blind, placebo-controlled study designed to assess the safety, tolerability, pharmacokinetics, and pharmacodynamics of SAD and MAD. The study includes healthy participants as well as individuals with T2DM, with treatment periods lasting up to six weeks. As of July 2025, the trial is listed as active and not recruiting, and results have not yet been posted [112].

### 5.12. PF-06954522

PF-06954522 is an oral small-molecule GLP-1R agonist developed by Pfizer (New York, NY, USA) in collaboration with Sosei Heptares (Tokyo, Japan). Specific preclinical data for PF-06954522 are limited in the public domain. However, the compound’s progression into Phase I clinical trials indicates that it has undergone initial preclinical evaluations to assess its pharmacological properties, efficacy, and safety profile.

In a randomized, double-blind, placebo-controlled Phase I trial (NCT06003777), healthy participants were enrolled into three cohorts and received single-ascending doses (SAD) under fasted or fed conditions. PK analyses showed dose-proportional exposure, with AUClast values of 1394–7503 ng·h/mL and Cmax up to 837.6 ng/mL in the fasted state. Tmax was 1–3 h, and t½ was 6–8.4 h, supporting once-daily dosing. The compound was well tolerated overall, with no deaths or serious AEs. The most common AEs were GI, particularly nausea (≤66.7%) and vomiting (≤87.5%), generally mild to moderate. Less frequent AEs included headache, dizziness, fatigue, and decreased appetite. No clinically relevant abnormalities were noted in labs, vital signs, or ECG parameters [113].

To further support formulation development, a subsequent open-label, multiple-period crossover study (NCT06393517) compared the single-dose pharmacokinetics of immediate-release versus modified-release formulations of PF-06954522 in healthy volunteers. Completed in September 2024, this trial aimed to characterize the absorption profiles and inform the selection of an optimized once-daily modified-release formulation [114]. In parallel, PF-06954522 has also been evaluated in a Phase I, randomized, double-blind, sponsor-open, placebo-controlled trial (NCT06279234) involving adult participants with T2DM. This study assessed the safety, tolerability, pharmacokinetics, and pharmacodynamics of multiple escalating oral doses in individuals with inadequately controlled T2D on background metformin therapy (Part A), with an optional exploratory arm in non-diabetic participants with obesity (Part B) [115]. Although both studies (NCT06393517 and NCT06279234) have been completed, their results have not yet been revealed.

## 6. Conclusions

Non-peptidic GLP-1R agonists represent a promising class of therapeutic agents for T2DM and obesity, offering the potential advantages of oral bioavailability and convenient dosing over traditional peptide-based GLP-1R agonists. Preclinical and early clinical data indicate that these compounds can effectively engage the GLP-1 receptor, elicit robust cAMP-mediated signaling, and induce glucose-lowering, insulinotropic, anorexigenic, and weight-reducing effects comparable to those of peptide analogues.

Pharmacokinetic profiles vary across molecules but generally support once- or twice-daily oral dosing, with dose-proportional systemic exposure, moderate to long elimination half-lives, and predictable absorption patterns. Some compounds exhibit limited central penetration, suggesting that peripheral receptor activation may suffice for therapeutic efficacy.

Safety profiles are largely consistent with the GLP-1R agonist class, with gastrointestinal adverse events—primarily nausea, vomiting, and diarrhea—being the most common. Early evidence of cardiovascular or metabolic benefits is encouraging, although long-term studies are needed to fully establish these outcomes.

Overall, non-peptidic GLP-1R agonists demonstrate a favorable balance between efficacy, pharmacokinetics, and safety, positioning them as viable alternatives to injectable peptide therapies. Future development should focus on minimizing GI side effects, and evaluating long-term clinical outcomes in large, diverse patient populations.

## Figures and Tables

**Table 1:** Approved Peptide-Based GLP-1 Receptor Agonists for Type 2 Diabetes and Obesity.

GLP-1R Agonist	Brand Name	Approval Year	Dose and Route of Administration
Type 2 Diabetes			
Exenatide	Byetta (Astra Zeneca)	2006	5 μg and 10 μg twice a day (SC)
Liraglutide	Victoza (Novo Nordisk)	2009	0.6 mg, 1.2 mg and 1.8 mg once a day (SC)
Exenatide ER	Bydureon (AstraZeneca)	2011	2 mg once a week (SC)
Lixisenatide	Adlyxin/Lyxumia (Sanofi)	2011	10 μg and 20 μg once a day (SC)
Dulaglutide	Trulicity (Eli Lilli)	2014	0.75 mg, 1.5 mg, 3 mg and 4.5 mg once a week (SC)
Semaglutide	Ozempic (Novo Nordisk)	2018	0.25 mg, 0.5 mg 1 mg and 2 mg once a week (SC)
	Rybelsus (Novo Nordisk)	2020	1.5 mg, 3 mg, 4 mg, 7 mg, 9 mg, 14 mg 25 mg and 50 mg once a day (PO)
Tirzepatide ^#^	Mounjaro (Eli Lilly)	2022	2.5 mg, 5 mg, 10 mg and 15 mg once a week (SC)
Obesity			
Liraglutide	Saxenda (Novo Nordisk)	2015	0.6 mg, 1.2 mg 1.8 mg, 2.4 mg and 3 mg once a day (SC)
Semaglutide	Wegovy (Novo Nordisk)	2021	0.25 mg, 0.5 mg 1 mg, 1.7 mg and 2.4 mg once a week (SC)
Tirzepatide ^#^	Mounjaro (Eli Lilly)	2024	2.5 mg, 5 mg, 10 mg and 15 mg once a week (SC)

Legend: The table summarizes currently approved peptide-based GLP-1 receptor agonists, indicating brand names, approval years, dosing regimens, and routes of administration for type 2 diabetes and obesity. ^#^ Tirzepatide is included as a dual GLP-1R/GIPR agonist. Abbreviations: GLP-1R—Glucagon-Like Peptide-1 Receptor; SC—subcutaneous; ER—extended release; PO—orally; Glucose-Dependent Insulinotropic Polypeptide Receptor.

**Table 2:** Pharmacological profile of oral small-molecule GLP-1R agonists.

Agonist	Developer	Mechanism/Binding Profile	Preclinical Findings	Clinical Development (Phase/NCT)	Key Efficacy Outcomes	Safety/Tolerability
Boc5	Institute of Materia Medica (Shanghai, China)	Orthosteric full agonist Biased signaling profile, no β-arrestins recruiting	Decreased blood glucose and HbA1c Body weight reduction	-	-	-
TT-OAD2	Eli Lilly & Co. (Indianapolis, IN, USA)	Orthosteric partial agonist Biased signaling profile, no β-arrestins recruiting	Insulinotropic effects	-	-	-
TTP-273	vTv Therapeutics (High Point, NC, USA)	Orthosteric partial agonist Biased signaling profile	Improved glycemic control Reduced food intake	Phase IIa (NCT02653599)	Modest HbA1c reduction and weight loss (150 mg once daily)	Favorable GI tolerability profile
Danuglipron (PF-06882961)	Pfizer (New York, NY, USA)	Orthosteric full agonist for cAMP signaling Partial activation of other pathways	Glucose-lowering Anorexigenic effects Cardioprotective effects	Phase IIb (NCT04707313)	Dose-dependent reductions in HbA1c and body weight (40–120 mg twice daily)	Mild GI AEs Asymptomatic liver enzyme elevations
Orforglipron (LY3502970, OWL833)	Eli Lilly (Indianapolis, IN, USA)/Chugai Pharmaceutical (Tokyo, Japan)	Orthosteric partial agonist Biased signaling profile, no β-arrestins recruiting	Glucose-lowering activity Reduced food intake	Phase III (NCT05869903)	Significant reductions in HbA1c dose-dependent weight loss (9–45 mg once daily)	GI AEs of mild to moderate intensity
HRS-7535	Shandong Suncadia Medicine (Lianyungang, Jiangsu, China)/Kailera Therapeutics (San Diego, CA, USA)	ND	ND	Phase II (NCT06250946)	HbA1c reductions dose-proportional body weight reduction (30–180 mg once daily)	Mild GI AEs
ECC5004 (AZD5004)	Eccogene (Shanghai, China)/AstraZeneca (Cambridge, UK)	Orthosteric full agonist Biased signaling profile, no β-arrestins recruiting	Enhanced insulin secretion Reduction in body weight gain	Phase II (NCT06579105)	Glucose-lowering efficacy and reduction in body weight (4–300 mg once daily)	Mild GI AEs
CT-996	Roche Holding AG (Basel, Switzerland)/Carmot Therapeutics, (Berkeley, Berkeley, CA, USA)	Orthosteric partial agonist Biased signaling profile, reduced β-arrestins recruiting	Improved glycemic control and insulin secretion Body weight regulation	Phase II (NCT07112872)	Body weight reduction (10–120 mg once daily)	Mild to moderate GI AEs
Aleniglipron (GSBR-1290)	Structure Therapeutics (Shanghai Shi, China)/ Gasherbrum Bio/ (San Francisco, CA, USA)	Orthosteric full agonist Biased signaling profile, no β-arrestins recruiting	Increase in insulin secretion and glucose clearance Reduction in food intake and body weight	Phase IIb (NCT06693843)	Reduced HbA1c, body weight and plasma glucose (45–120 mg once daily)	Mild to moderate GI AEs
ID110521156	Yunovia (Gyeonggi-do, Republic of Korea)/ Ildong Pharmaceutical (Seoul, Republic of Korea)	Orthosteric full agonist Partial/no activation of other pathways	Reduced plasma glucose levels and enhanced insulin secretion Reductions in food intake and body weight	Phase I (NCT06635226)	In progress	Mild GI AEs
XW-014	Hangzhou Sciwind Biosciences (Zhejiang Sheng, China)	ND	Reduced blood glucose Reduced food intake and body weight	Phase I (NCT05579314)	In progress	In progress
PF-06954522	Pfizer (New York, NY, USA)/Sosei Heptares (Tokyo, Japan)	ND	ND	Phase I (NCT06279234)	ND	Mild to moderate GI AEs

Legend: This table summarizes key pharmacological properties, preclinical findings, and clinical development stages of oral small-molecule GLP-1 receptor agonists. Information includes mechanism of action, binding profile, efficacy outcomes, and safety/tolerability signals as reported in published studies and registered clinical trials. Abbreviations: GI—gastro-intestinal; HbA1c—glycated hemoglobin; ND—not disclosed; AEs—adverse effects; NCT—National Clinical Trial.

## Data Availability

All data generated or analyzed during this study are included in this published article.
